# Sustainable Ultrasound-Assisted Extraction and Recovery of Rare Earth Elements from Oil and Gas Drill Cuttings

**DOI:** 10.1021/acsomega.5c11240

**Published:** 2026-01-21

**Authors:** Klaiani Bez Fontana, Eduardo Sidinei Chaves, Caroline Gonçalves, Elias Paiva Ferreira, Sidney José Lima Ribeiro, Rennan Geovanny Oliveira Araujo, Tatiane de Andrade Maranhão

**Affiliations:** 1 Departamento de Química, Campus Trindade, 28117 Universidade Federal de Santa Catarina, 88040-900 Florianópolis, Santa Catarina, Brazil; 2 Departamento de Química Analítica, Físico-química e Inorgânica, Instituto de Química, 28108 Universidade Estadual Paulista, 14800-060 Araraquara, São Paulo, Brazil; 3 Departamento de Química Analítica, Instituto de Química, Campus Universitário de Ondina, Universidade Federal da Bahia, 40170-115 Salvador, Bahia, Brasil

## Abstract

Drill cuttings (DC) are mainly composed of fragmented rocks that are produced during the drilling of oil and natural gas wells. The rocks and minerals present in DC are sources of several chemical elements, including economically valuable elements, such as rare earth elements (REEs). In this paper the sustainability and advantages of the ultrasound-assisted extraction were applied for the extraction and recovery of REEs from DC. To determine the optimal extraction conditions, a full factorial and Doehlert matrix design was employed. The DC were characterized by XRD and EDXRF, and the REEs concentrations were determined by ICP-MS. The REEs recovery was performed by precipitation with oxalic acid. The analyzed DC samples were primarily composed of aluminosilicate minerals, the α-quartz, calcite, albite, and muscovite being the most common phases. The EDXRF analysis confirms the predominance of silicate and aluminosilicate rock-forming minerals and accounts for the significant content of Fe, Ca, Na, and Mg. The optimal extraction condition was achieved using HNO_3_ at 7.0 mol L^–1^ as extractor and ultrasound-assisted extraction at 80 °C for 60 min. The efficiency of extraction in the DC analyzed was high (for La and Ce, >82%), and after precipitation, good recoveries were achieved, especially for La and Ce (>90%). Thus, the proposed ultrasound-assisted extraction process proved to be suitable for the recovery of these elements from DC, contributing to sustainable development and the circular economy.

## Graphical abstract

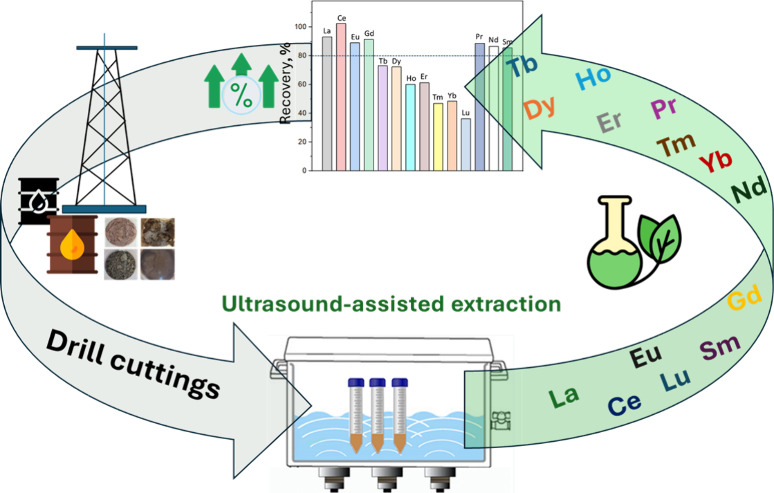

## 1 Introduction

The exploration of oil and gas in offshore petroleum basins is an activity with significant impacts on the economy and the environment. The petroleum exploration phase is one of the most environmentally impactful steps of production, mainly due to the large amount of waste generated.^1^ Drill cuttings (DC) are one of the main wastes produced during the drilling of oil and natural gas exploration, development, and production wells; these wastes represent high environmental concern. This waste is mainly composed of rock fragments, produced by the drill bit during well drilling, and drilling fluid, which is required for transporting these rock fragments to the surface. During the well drilling, different geological phases are encountered, which contribute to the production of DC with distinct characteristics/composition.^2–4^ The rocks and minerals present in DC are sources of several chemical elements, including economically valuable ones such as rare earth elements (REEs).

The REEs have great economic value due to their wide application in various industrial sectors, especially in high technology.^5,6^ However, because of their increasing use, studies have reported a significant negative impact to the environment caused by the anthropogenic input of REEs.^7,8^ The recovery of valuable elements from waste is an environmentally friendly alternative source of these elements that contributes to meeting the high industry demand for REEs. Additionally, waste mining is an alternative to natural resource for REEs extraction that is in accordance with the circular economy and the sustainable development.^9,10^

Several strategies have been utilized for the recovery of REEs from waste, such as solvent extraction, electrolytic extraction, ion exchange, and biological processes.^11–13^ To improve the recovery efficiency of REEs, mineral acid solutions (such as HCl, H_2_SO_4_, and HNO_3_) have been commonly applied as extractor solvent. Microwave and ultrasound-assisted strategies and the conventional mechanical agitation have been proposed for extraction and recovery of REEs from different matrices.^14^ However, ultrasound-assisted extraction (UAE) is still little explored for REEs recovery, and it has advantages such as simplicity, low cost, and minimum energy consumption, promoting efficient extraction using diluted acids. The higher efficiency of UAE when compared to the conventional mechanical agitation systems has already been demonstrated for REEs extraction in environmental samples.^14,15^ The ultrasound waves promote cavitation in the extractor medium. In this phenomenon microbubbles of cavitation are produced, go through cycles of expansion and compression, increasing in size until collapse.^16^ During the collapse of the microbubbles, localized zones of high temperature and pressure are produced, generally increasing the extraction efficiency.^17^ Thus, the use of UAE has been increasing in recent years because this technique aligns with the principles of green chemistry and enables the development of more sustainable analytical procedures.^18–20^ The UAE has been effectively applied for extraction/recovery of REEs elements from wastes such as fluorescent lamps,^21^ carbonatite rocks,^22^ permanent magnet,^23^ and phosphogypsum.^24^ However, the extraction and recovery of REEs in a complex sample such as drill cutting from oil and gas drilling wells have not been reported.

In this work, the effectiveness and sustainability advantages of the UAE were applied for the extraction and recovery of REEs from DC. The REEs extraction process was optimized by using a full factorial and Doehlert matrix design to determine the extraction conditions. This multivariate approach allows a more comprehensive evaluation of the effects and interactions of the extraction parameters. Additionally, the REEs were selectively recovered through precipitation with oxalic acid.

## 2 Experimental Section

### 2.1 Instrumental

The samples were dried in an oven model 238 (Biomatic, Porto Alegre, Brazil). For the extraction procedure, an analytical balance ATX224 (Shimadzu, Brazil), an ultrasonic bath 30LTS, model T50, operating at frequency of 40 kHz and 600 W (Thornton, São Paulo, Brazil), and a centrifuge Q222TM216 (Quimis, São Paulo, Brazil) were used. The REEs concentration in the extracts and DC samples (total concentration) were determined in an Elan 6000 inductively coupled plasma mass spectrometer (PerkinElmer-Sciex, Thornhill, Canada). The instrument was equipped with platinum sampler and skimmer cones, and high-purity argon (99.996%) was employed as the plasma and nebulizer gas (White Martins, São Paulo, Brazil). For the measurements, the sample introduction was performed by a pneumatic crossflow nebulizer coupled to a Scott type double pass spray chamber. The instrumental operating conditions were radiofrequency power of 1100 W, nebulizer gas flow of 1.1 L min^–1^, and lens voltage of 9.5 V. The monitored REEs isotopes were ^138.9^La, ^139.9^Ce, ^152.9^Eu, ^157.9^Gd, ^158.9^Tb, ^163.9^Dy, ^164.9^Ho, ^165.9^Er, ^168.9^Tm, ^173.9^Yb, ^174.9^Lu, ^151.9^Sm, ^140,9^Pr, and ^141.9^Nd. The elemental composition of the DC was determined in an energy-dispersive X-ray fluorescence spectrometer model S2 Ranger, equipped with a palladium tube and Si–Li detector (Bruker, Germany). For the mineralogical analysis, a MiniFlex 600 X-ray diffraction spectrometer (Rigaku, Japan) equipped with a copper radiation source was used.

### 2.2 Reagents and Sample

All the reagents used were at least of analytical grade. Deionized water, with a resistivity of 18.2 MΩ cm, was obtained by a Milli-Q system (Millipore, USA). Nitric acid 65% m m^–1^ (Vetec, Rio de Janeiro), purified by subboiling double distillation, was used for leaching of REEs. Sodium hydroxide (Neon, São Paulo) and oxalic acid (Fluka, Germany) were used for precipitation and recovery of REEs. For ICP-MS analysis, REEs multielement standard solution (Sigma-Aldrich, Switzerland) and rhodium (Rh) (Sigma-Aldrich, Switzerland), internal standard at a concentration of 5.0 μg L^–1^, were used for calibration purposes.

The DC samples were kindly provided by a Brazilian oil and gas company. The samples come from oil well collected at different depths: 3016 m (A1), 3157 m (A2), 3190 m (A3), 3278 m (A4), 3366 m (A5), 3453 m (A6), and 3541 m (A7). The dried samples were manually homogenized using an agate mortar and pestle and sieved in 100 μm thick nylon mesh.

### 2.3 Ultrasound-Assisted Extraction

The ultrasonic bath employed does not allow for changes to frequency or power. First, univariate evaluation of the influence of the sample mass (25, 50, 75, 110, 125, and 150 mg) on the extraction of REEs was carried out. Subsequently, complete factorial design 2^3^ was applied to define the significance of UAE parameters (variables). The evaluated extraction parameters were time (5–55 min), extraction temperature (25–75 °C), and HNO_3_ concentration (2.8–11.2 mol L^–1^). Afterward, for the statistically significant variables, ultrasound time (30–90 min) and extraction temperature (40–80 °C), a Doehlert design was applied to obtain optimal extraction conditions. For REEs extraction, the DC samples were weighed in polypropylene tubes, added to 2 mL of the extractor (HNO_3_), and exposed to the UAE at controlled temperature. Then, using deionized water, the volume was filled up to 10 mL, the mixtures were centrifuged for 10 min at 3000 rpm, the supernatant extract containing the REEs was separated, and the REEs were determined by ICP-MS. The multiple response (MR) was considered for the multivariate UAE optimization.^25^ The MR function was obtained by the combination of the individual REE concentration obtained in each experiment divided by the maximum concentration of the repetitive REE obtained for the set of experiments, as shown in eq 1,

1 $$\mathrm{MR}=\left({\left[\mathrm{La}\right]}_{i}/{\left[\mathrm{La}\right]}_{\max}\right)+\left({\left[\mathrm{Ce}\right]}_{i}/{\left[\mathrm{Ce}\right]}_{\max}\right)+\left({\left[\mathrm{Eu}\right]}_{i}/{\left[\mathrm{Eu}\right]}_{\max}\right)+\left({\left[\mathrm{Gd}\right]}_{i}/{\left[\mathrm{Gd}\right]}_{\max}\right)+\left({\left[\mathrm{Tb}\right]}_{i}/{\left[\mathrm{Tb}\right]}_{\max}\right)+\left({\left[\mathrm{Dy}\right]}_{i}/{\left[\mathrm{Dy}\right]}_{\max}\right)+\left({\left[\mathrm{Ho}\right]}_{i}/{\left[\mathrm{Ho}\right]}_{\max}\right)+\left({\left[Er\right]}_{i}/{\left[Er\right]}_{\max}\right)+\left({\left[\mathrm{Tm}\right]}_{i}/{\left[\mathrm{Tm}\right]}_{\max}\right)+\left({\left[\mathrm{Yb}\right]}_{i}/{\left[\mathrm{Yb}\right]}_{\max}\right)+\left({\left[Lu\right]}_{i}/{\left[Lu\right]}_{\max}\right)+\left({\left[\mathrm{Sm}\right]}_{i}/{\left[\mathrm{Sm}\right]}_{\max}\right)+\left({\left[\mathrm{Pr}\right]}_{i}/{\left[\mathrm{Pr}\right]}_{\max}\right)+\left({\left[\mathrm{Nd}\right]}_{i}/{\left[\mathrm{Nd}\right]}_{\max}\right)$$

where [REE]_i_ represents the concentration of the REEs in each individual experiment, and [REE]_max_ is the highest concentration of the respective element obtained across all experiments.

The REEs were quantified by ICP-MS using a calibration range from 1 to 60 μg L^–1^, and Rh (5 μg L^–1^) as an internal standard. The detection (LoD) and quantification (LoQ) limits were, respectively, established by three and ten times the standard deviation of 10 consecutive blank measurements, divided by the slope of the respective calibration curve. The obtained LoQs values ranged from 2.0 to 8.5 μg kg ^–1^, which were suitable for the analysis. Total REEs concentrations in the DC samples were determined by ICP-MS after microwave-assisted acid decomposition as established in previous work.^26^ The experimental data were processed using Statistica software version 6.0 (StatSoft, Tulsa, USA) and Origin 6.0 (OriginLab Corporation, Northampton, MA).

The REEs extraction efficiency, expressed in percentage (%), was determined by comparing the concentration of the extracted REEs with the total concentration of the REEs determined after microwave-assisted acid digestion (eq 2),

2 $$\mathrm{REE extraction}\left(\%\right)=\left({\mathrm{REE}}_{\left(\mathrm{extracted}\right)}/{\mathrm{REE}}_{\left(\mathrm{total}\right)}\right)\times 100$$

where REE _(total)_ represents the total concentrations of each rare earth element (La, Ce, Eu, Gd, Tb, Dy, Ho, Er, Tm, Yb, Lu, Sm, Pr, and Nd) obtained after microwave-assisted decomposition, and REE_(extracted)_ corresponds to the concentration of each extracted element in solution.

### 2.4 REEs Precipitation and Recovery

The assessment of REEs recovery was performed by precipitation of the elements with oxalic acid. The REEs precipitation procedure was adapted from Ippolito et al. (2017).^27^ In the extracts of the DC containing the REEs, an aliquot of oxalic acid was added up to a final concentration of 10% (w v^–1^) and, subsequently, the pH was adjusted to 2.5. For a complete precipitation, the solution was kept at room temperature for 24 h, and then the REEs precipitated were separated by centrifugation. The efficiency of precipitation was obtained by considering the concentration of extracted REEs in solution prior to and after the precipitation step (eq 3).

3 $$\mathrm{REE precipitation}\left(\%\right)=\left(\left({\mathrm{REE}}_{\left(\mathrm{extracted}\right)}-{\mathrm{REE}}_{\left(\mathrm{AP}\right)}\right)/{\mathrm{REE}}_{\left(\mathrm{extracted}\right)}\right)\times 100$$

where REE_(extracted)_ corresponds to the concentration of each extracted element determined in solution prior to the REEs precipitation, and REE_(AP)_ corresponds to the remaining concentration of REEs in the supernatant solution determined after precipitation step.

Thus, to determine the element recoveries, the REEs concentration in the supernatant after precipitation step (REE_(AP)_) and the total REEs concentration in DC (REE_(total)_) were considered and the % REE recovery by precipitation was obtained by eq 4.

4 $$\mathrm{REE recovery}\left(\%\right)=\left(\left({\mathrm{REE}}_{\left(\mathrm{extracted}\right)}-{\mathrm{REE}}_{\left(\mathrm{AP}\right)}\right)/{\mathrm{REE}}_{\left(\mathrm{total}\right)}\right)\times 100$$

The main reaction for the extraction and recovery of the REEs is illustrated by reactions 5 and 6.

Extraction:

5 $${\mathrm{REE}}_{2}O_{3}\left(s\right)+6{\mathrm{HNO}}_{3}\left(\mathrm{aq}\right)\to 2{\mathrm{REE}\left({\mathrm{NO}}_{3}\right)}_{3}\left(\mathrm{aq}\right)+3H_{2}O\left(l\right)$$

Precipitation:

6 $${2\mathrm{REE}\left({\mathrm{NO}}_{3}\right)}_{3}\left(\mathrm{aq}\right)+3H_{2}C_{2}O_{4}\left(\mathrm{aq}\right)\to {{\mathrm{REE}}_{2}\left(C_{2}O_{4}\right)}_{3}\left(s\right)+6{\mathrm{HNO}}_{3}\left(\mathrm{aq}\right)$$

REE are the rare earth elements (La, Ce, Eu, Gd, Tb, Dy, Ho, Er, Tm, Yb, Lu, Sm, Pr, Nd).

### 2.5 Characterization Analysis

The mineralogical characterization of the DC samples was performed in an X-ray diffraction (XRD) spectrometer using glass sample holders. The analyses were performed at 2° min^–1^ from 5 to 90° and a step of 0.02°. To identify the phases, the HighScore Plus software was used. Phase quantification was carried out using the Rietveld method^28^ with analysis performed in GSAS-II software.^29^

The determination of the elemental composition of samples and precipitated REEs was performed by energy dispersive X-ray fluorescence (EDXRF), using sample preparation as pressed pallet samples. To obtain pellets, a mass of 1.4 g of sample and 0.6 g of boric acid were used as a binder. The samples and binder were homogenized in agata mortar and pressed in a manual hydraulic at approximately 2 × 10^2^ MPa for 2 min.

## 3 Results and Discussion

### 3.1 Drill Cuttings Characterization

Semiquantitative determination of the main chemical elements in the DC samples was performed using X-ray fluorescence (Figure 1), and results are presented in Table S1. As observed, the samples are primarily composed of aluminosilicate minerals with high Si and Al content. Elements such as Ca, Na, K, Mg, and Fe also make up the primary mineral composition of the DC samples and, along with Si and Al, are commonly found in reservoir rocks.^30^

**Figure 1:**
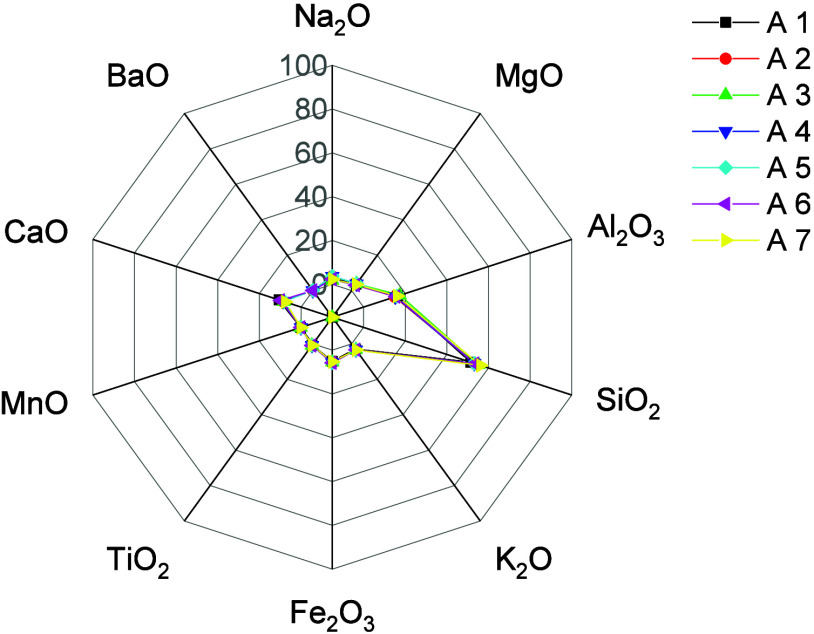
Elemental composition in drill cuttings samples (A1–A7) determined by X-ray fluorescence.

Mineral phase analysis was conducted using XRD, and the content of each identified mineral phase was estimated through Rietveld refinement. Figure 2 shows the representative refined XRD pattern of the DC sample, while Table 1 summarizes the results of mineral phase fractions as determined by the Rietveld refinement. Rwp values below 10% (3.27–4.44%) generally suggest a reasonable match between the calculated and experimental XRD data, indicating a good level of refinement accuracy. GoF values, on the other hand, indicate how well the model explains the data, with ideal values being closer to 1 (Toby, 2006).^31^ In this data set, GoF values range from 1.84 to 2.45, showing that the model provides an adequate fit to the data, although it does not completely describe all data details. Slightly higher GoF values are acceptable for complex samples such as these mixed-mineral samples, considering their compositional variability, complex microstructural characteristics, and the possible presence of unidentified minor phases. Overall, the Rwp and GoF values support the reliability of the mineral phase composition results for the DC samples. All the analyzed CP samples exhibited a similar mineral composition, primarily consisting of α-quartz (SiO_2_, ICSD-83849),^40^ calcite (CaCO_3_, ICSD-16710),^28^ calcian albite ((Na_1–x_Ca_x_)­AlSi_3_O_8_, ICSD- 34917),^32^ and muscovite (KAl_2–x_Fe_x_(AlSi_3_O_10_)­(OH)_2_, COD-1100010).^33^ Minor phases included minerals such as biotite (K­(Mg,Fe)_3_­AlSi_3_O_10_­(OH)_2_, COD-9001266)^34^ and kaolinite (Al_2_Si_2_O_5_(OH)_4_, COD-1011045.^35^ The XRD phase analysis is consistent with the EDXRF composition results, confirming the predominance of silicate and aluminosilicate rock-forming minerals and accounting for the significant contents of Fe, Ca, Na, and Mg.

**Figure 2:**
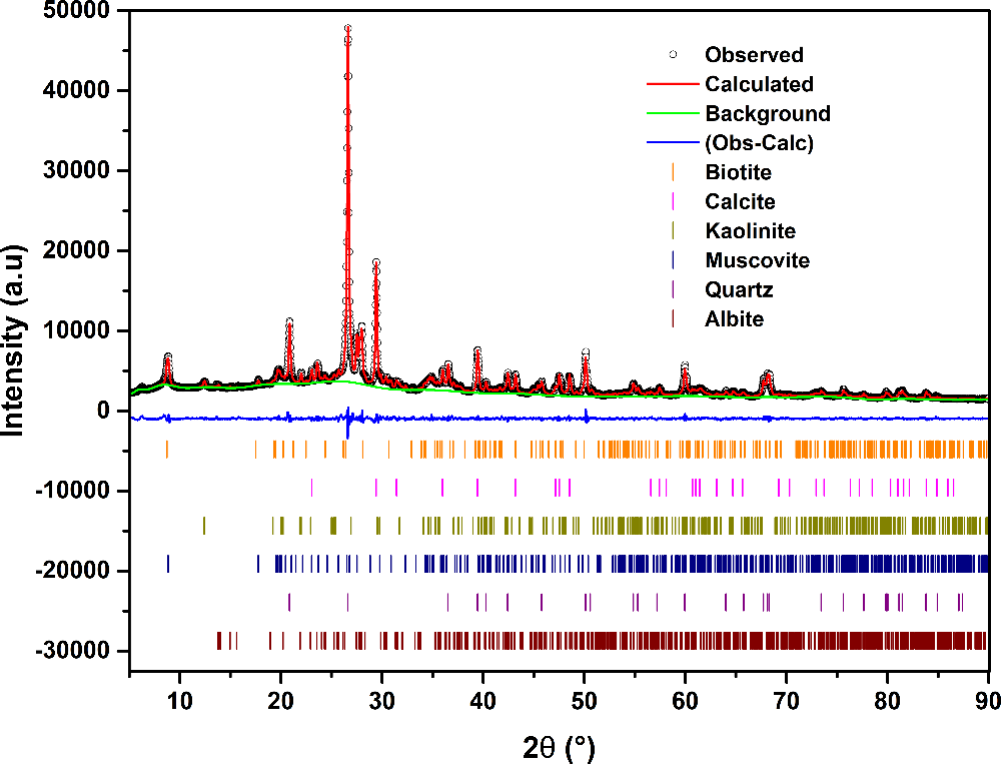
Rietveld refinement plot of X-ray diffraction (XRD) data for a multiphase mineral sample (A2). The observed (obs) and calculated (calc) patterns are shown along with the background (bkg) and difference (diff) curves. Vertical markers indicate the peak positions of identified mineral phases, including biotite, calcite, kaolinite, muscovite, quartz, and albite.

**Table 1:** Semiquantitative Composition of Mineral Phases in DC Samples, As Determined by Rietveld Refinement^a^

sample	α-quartz (%)	calcite (%)	albite (%)	muscovite (%)	biotite (%)	kaolinite (%)	Rwp (%)	GOF
A1	40.6 ± 0.3	19.2 ± 0.3	19.6 ± 0.2	11.8 ± 0.2	6.1 ± 0.2	2.6 ± 0.1	4.44	2.38
A2	33.6 ± 0.6	16.6 ± 0.3	20.6 ± 0.6	18.7 ± 0.5	5.5 ± 0.3	5.0 ± 0.3	3.27	1.84
A3	41.5 ± 0.7	13.9 ± 0.4	21.0 ± 0.7	13.2 ± 0.6	5.6 ± 0.4	5.0 ± 0.4	3.89	2.18
A4	34.8 ± 0.4	17.0 ± 0.3	17.6 ± 0.4	19.4 ± 0.4	6.2 ± 0.3	5.0 ± 0.4	3.42	1.93
A5	43.9 ± 0.8	15.7 ± 0.4	16.3 ± 0.7	13.5 ± 0.7	6.5 ± 0.4	4.2 ± 0.5	3.73	2.07
A6	41.6 ± 0.6	15.3 ± 0.4	18.6 ± 0.5	10.6 ± 0.6	6.0 ± 0.3	8.0 ± 0.4	3.46	2.06
A7	41.6 ± 0.7	11.6 ± 0.3	21.5 ± 0.5	13.3 ± 0.6	5.3 ± 0.4	6.8 ± 0.4	4.19	2.45

a Values represent the relative mass fractions of each mineral phase, including α-quartz, calcite, albite, muscovite, biotite, and kaolinite. The statistical parameters Rwp (weighted profile *R*-factor) and GoF (goodness of fit) are included to indicate the quality of the refinement for each sample.

### 3.2 Optimization of Ultrasound-Assisted Extraction

Considering that the efficiency of acid extraction of metals by ultrasound depends on parameters related to the sample’s characteristics, composition of extractor solution and the ultrasound system used the following variables were selected for the optimization step:^15,36^ sample mass, extraction time and temperature, and the concentration of HNO_3_ in the extractor solution. First, the the sample mass effect on the REEs extraction efficiency was univariately evaluated (Figure 3).

**Figure 3:**
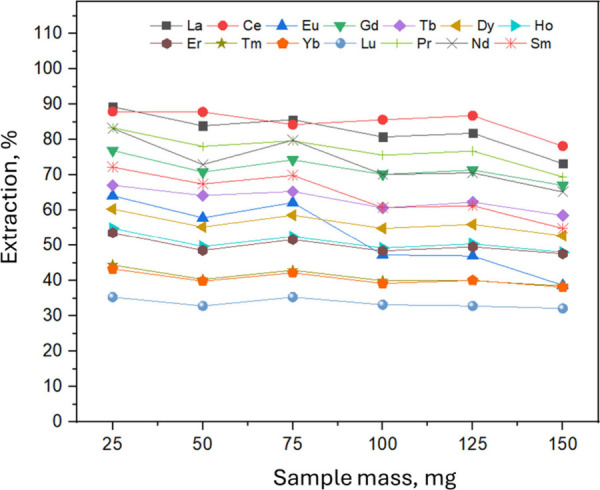
Effect of the sample mass on ultrasound-assisted extraction of REEs in drill cuttings. Ultrasound time, 50 min; extraction temperature, 75 °C; 2 mL of extractor HNO_3_ = 2.8 mol L^–1^.

The assessment of significant difference was performed by statistical analysis, applying analysis of variance (ANOVA) followed by Tukey’s test, with a confidence level of 95%. The results indicated that the REEs concentration obtained using 25, 50, and 75 mg masses did not present significant differences between them, while higher masses showed significant differences at the 95% confidence level. These data show that at low liquid/solid ratios, the mass transfer coefficient is affected by limiting the leaching efficiency of REEs.^37^ The researchers found that by increasing the liquid/solid ratio, the REEs leaching increases until reaching a critical point where the liquid/solid ratio increasing does not affect the leaching. Thus, considering the fixed volume of extractor solvent (2 mL), 75 mg of sample was selected for subsequent optimizations, since this condition contributes to the effectiveness of REEs extraction in drill cuttings.

Subsequently, a complete factorial design 2^3^ was performed to identify the significance of the variables (*p* < 0.05) and their interactions in the selected intervals.^38^ The center point was added in triplicate to estimate the experimental error and to check the curvature. The MR function, calculated according to eq 1, was used as an analytical response.^25^ The matrix of the complete factor design 2^3^ and the calculated MR are presented in Table 2.

**Table 2:** Matrix of 2^3^ Full Factorial Design with a Center Point and the Respective MR for Ultrasound-Assisted Extraction of REEs in Drill Cuttings^a^

experiment	HNO_3_, mol L^‑1^	time, min	temperature, °C	MR
1	2.8 (−1)	5 (−1)	25 (−1)	6.26
2	11.2 (1)	5 (−1)	25 (−1)	6.63
3	2.8 (−1)	55 (1)	25 (−1)	10.25
4	11.2 (1)	55 (1)	25 (−1)	10.60
5	2.8 (−1)	5 (−1)	75 (1)	11.75
6	11.2 (1)	5 (−1)	75 (1)	12.42
7	2.8 (−1)	55 (1)	75 (1)	12.91
8	11.2 (1)	55 (1)	75 (1)	13.10
9	7.0 (0)	30 (0)	50 (0)	12.10
10	7.0 (0)	30 (0)	50 (0)	12.03
11	7.0 (0)	30 (0)	50 (0)	13.00

a The coded values of the variables are presented in parentheses.

As observed, the lowest effect values of the responses were obtained for experiments 1 and 2, connected with the shorter ultrasound extraction time (5 min) and lower temperature (25 °C). However, in experiment 1 the concentration of HNO_3_ is the lowest level (2.8 mol L^–1^) and in experiment 2 the highest level (11.2 mol L^–1^). These results indicated the importance of the ultrasound time and temperature for the extraction of the REEs using an ultrasonic bath. The significance of the variables and their interactions was verified by analyzing ANOVA (analysis of variance) and Pareto’s chart using a confidence level of 95%. In Figure 4, Pareto’s chart for the proposed factorial design is shown.

**Figure 4:**
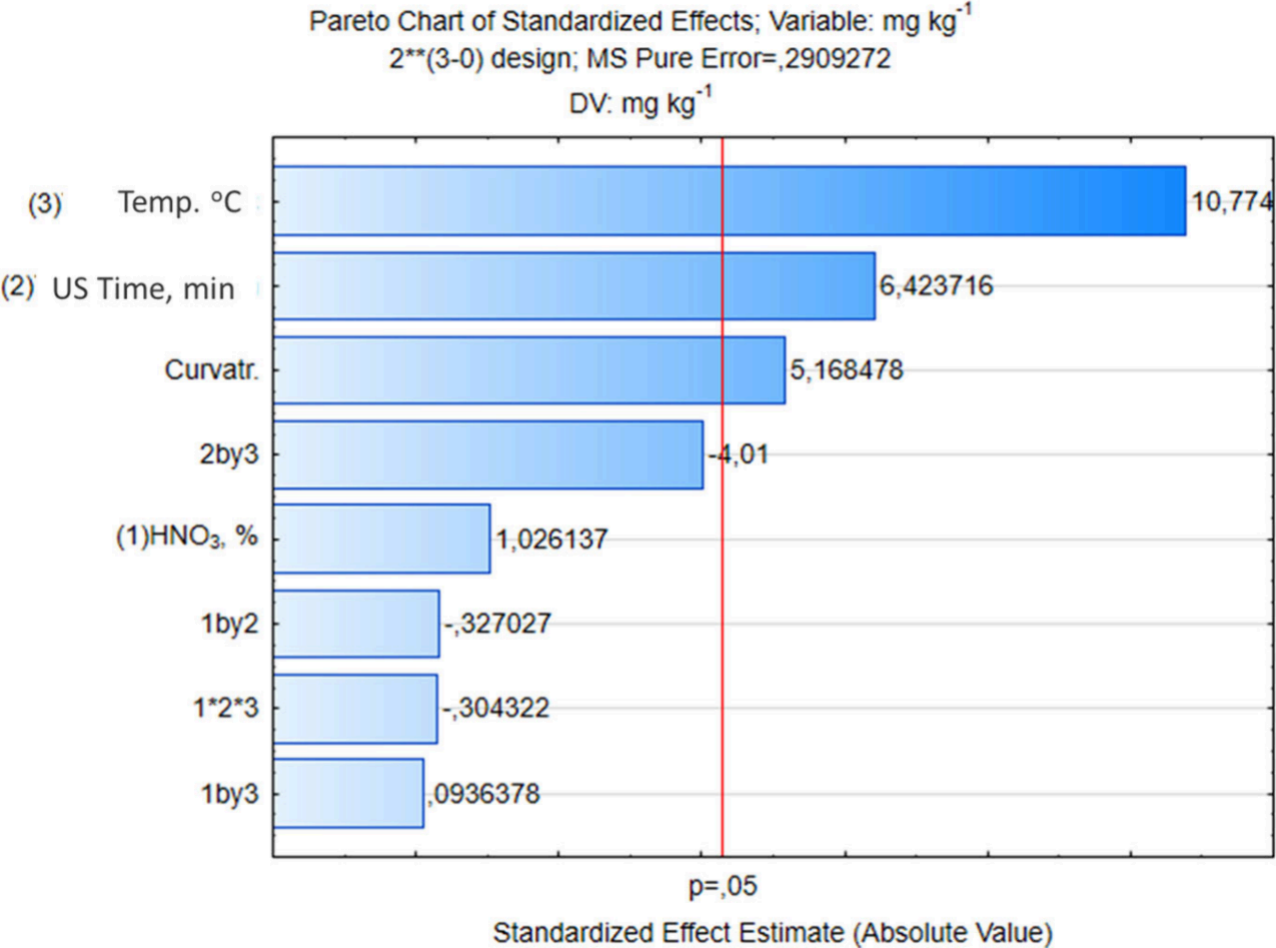
Pareto’s chart for 2^3^ full factorial design optimization of ultrasound-assisted extraction of REEs from drill cuttings.

It is observed that extraction temperature was the most significant variable (*p* < 0.05), followed by ultrasound time, both with positive effects on the REE extraction. Therefore, the increase in temperature and ultrasound time from lower to upper levels improves the effect of MR for REE extraction in the evaluated DC. The concentration of HNO_3_, as well as the interactions between the variables, did not have a significant effect on the evaluated levels. The curvature, showed to be statistically significant (*p* < 0.05) with a positive effect, indicates the existence of a region of maximum response between the evaluated lower and upper levels. The next experiments were performed using the extractor HNO_3_ 7.0 mol L^–1^; although the acid concentration did show a significant effect, the positive value may suggest that higher concentration benefits the REEs extraction. Thus, a response surface methodology (MSR), using a Doehlert design (Table 3), was applied to determine the optimal extraction conditions, considering the significant variables: ultrasound time and extraction temperature. The MR function (eq 1) was also used as a response to the proposed optimization.

**Table 3:** Doehlert Design Matrix and the MR for Ultrasound-Assisted Extraction of REEs in Drill Cuttings, Using HNO_3_ at 7.0 mol L^–1^ as the Extractor Solvent^a^

experiment	time, min	temperature, °C	MR
1	90 (1.0)	60 (0.0)	12.45
2	70 (0.5)	80 (0.866)	13.88
3	10 (−1.0)	60 (0.0)	9.43
4	30 (−0.5)	40 (−0.866)	7.43
5	70 (0.5)	40 (−0.866)	9.11
6	30 (−0.5)	80 (0.866)	12.83
7	50 (0.0)	60 (0.0)	12.59
8	50 (0.0)	60 (0.0)	12.75
9	50 (0.0)	60 (0.0)	11.35

a The coded values of the variables are presented in parentheses.

As observed, the lowest value of the MR was obtained in experiment 4, where the extraction of REEs was performed at temperature of 40 °C and 30 min of ultrasound extraction time, while the highest MR values were found in experiment 2, with a temperature of 80 °C and 70 min of ultrasound extraction. The corresponding response surface for the fitted quadratic model is shown in Figure 5.

**Figure 5:**
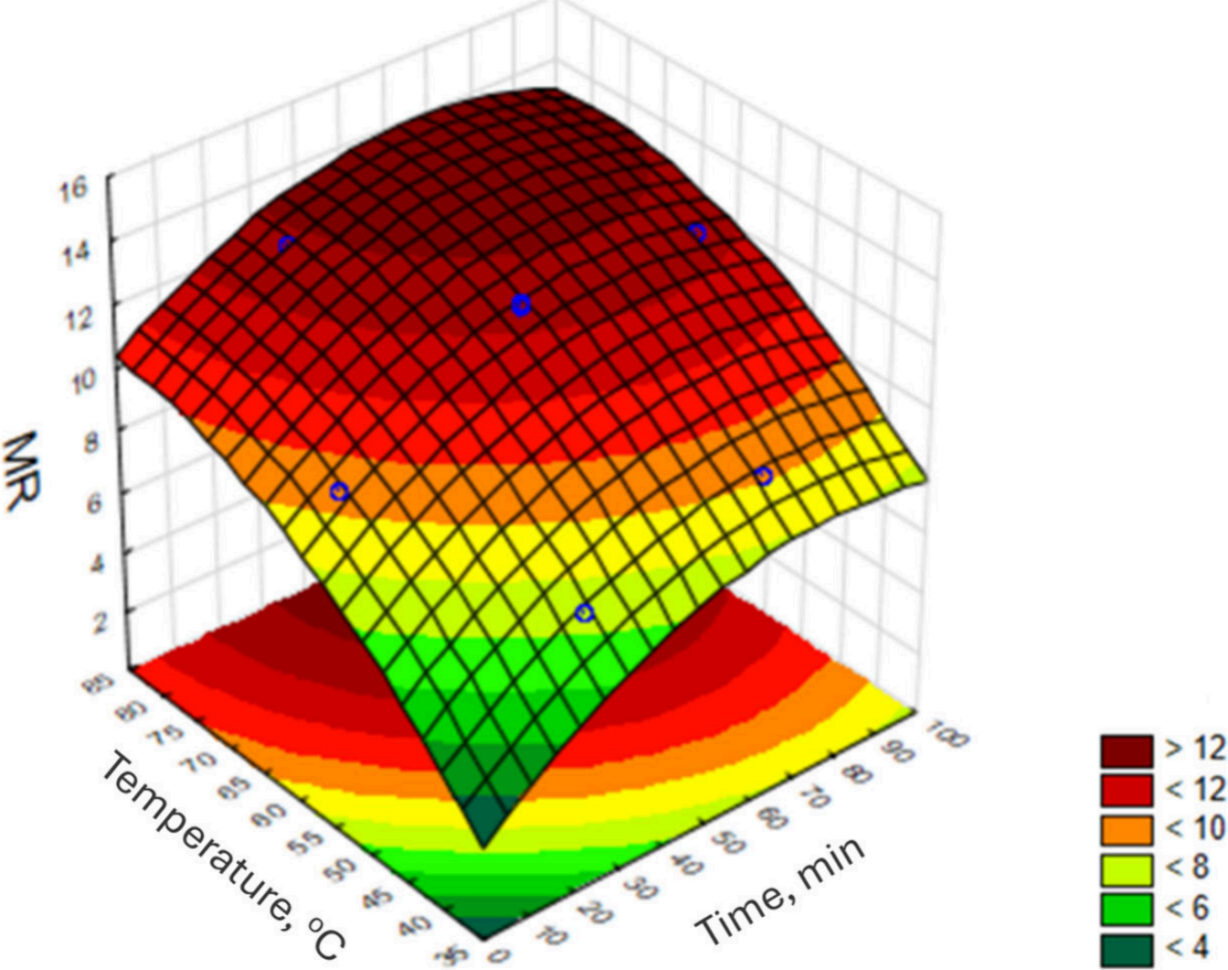
Response surface for Doehlert design optimization for ultrasound-assisted extraction of REEs from drill cuttings.

The quality of the adjusted quadratic model was evaluated by ANOVA. The model did not present a lack of fit, with a *p*-value of 0.88 (*p* > 0.05), indicating that the proposed model was satisfactory to describe the results for the Doehlert design with a confidence level of 95%. The correlation coefficient of 0.96 indicates a strong correlation of the predicted data to the experimental values. In Figure 5, it is possible to observe the existence of a region of maximum response. Accordingly, the optimal extraction condition was established with HNO_3_ at 7.0 mol L^–1^ as extractor solvent and ultrasound-assisted extraction at 80 °C for 60 min.

### 3.3 REEs Ultrasound-Assisted Extraction and Recovery

The optimized condition for the REEs extraction from DC using UAE (75 mg sample, HNO_3_ 7 mol L^–1^, 60 min ultrasound, and temperature of 80 °C) was applied to seven DC samples. The percentages of REEs extraction in each sample are listed in Table 4. To obtain the REEs extraction efficiency, the concentrations of the extracted REEs were compared with the total concentration of REEs determined by ICP-MS after microwave-assisted acid digestion (Table S2).

**Table 4:** Percentage of REEs Extraction in Drill Cuttings Using Ultrasound-Assisted Extraction

	extraction, %						
REE	A1	A2	A3	A4	A5	A6	A7
La	90.2	84.5	82.6	96.7	99.2	98.4	>99.9
Ce	>99.8	>99.8	93.5	>99.8	>99.9	>99.10	>99.7
Eu	75.6	80.3	>99.4	98.8	>99.4	96.7	>99.3
Gd	78.2	88.0	74.9	>99.5	97.8	94.9	>99.4
Tb	66.7	81.1	66.8	92.2	85.7	83.3	97.7
Dy	58.0	73.5	59.2	81.8	76.6	73.3	86.8
Ho	51.6	64.1	51.5	70.9	67.3	63.2	73.3
Er	51.5	59.0	50.1	64.4	62.1	59.5	69.6
Tm	40.5	45.7	37.2	52.8	51.6	47.5	50.6
Yb	41.1	43.2	36.3	47.4	45.3	44.1	49.4
Lu	32.6	32.7	28.5	35.3	35.9	37.1	35.7
Pr	79.3	87.0	80.8	92.2	93.4	88.0	>99.7
Nd	65.5	72.7	79.7	84.6	80.7	78.0	96.2
Sm	77.4	85.5	79.8	>96.5	>96.6	96.9	>99.7

Table 4 shows that the UAE provided a high extraction efficiency for La and Ce in all of the samples analyzed (82%). Among the REEs present in the DC, La and Ce showed the highest concentrations, with approximately 40 and 75 mg kg^–1^, respectively, and therefore a greater possibility for recovery. Sadeghi et al. (2020) applied ultrasound for La extraction in FCC catalysts. The authors also have demonstrated that UAE has been provided with a high extraction efficiency for La (97%), which corresponds to an increase of 27% when compared to mechanical agitation.^14^ Gatiboni et al. (2020) described the REEs extraction in carbonatite rocks using HNO_3_ (3% v v^–1^) and ultrasonic bath. The authors report that considering the sum of the extracted REEs, the system using ultrasonic bath provides a more efficient extraction (approximately 90%) while with mechanical agitation the extraction efficiency was only 60%.^15^

Considering the high percentage of REEs extraction obtained, mainly from La and Ce, the recovery of REE by using precipitation in the form of REE_2_(C_2_O_4_)_3_ was evaluated. After the UAE, the extracted REEs were directly precipitated from the sample extract by adding oxalic acid. The total concentration of REEs, along with the respective extracted and precipitated concentrations, as well as the precipitation and recovery percentages, is presented in Table 5.

**Table 5:** Total, Extracted, and Precipitated Concentrations and the Respective Precipitation Efficiency and Recoveries of REEs from Drill Cuttings^a^

	total, mg kg^‑1^	extracted, mg kg^‑1^	precipitated, mg kg^‑1^	precipitation efficiency, %	recovery, %
La	47.36 ± 0.69	42.70 ± 0.33	42.32 ± 0.08	99.1 ± 0.2	90.0
Ce	91.54 ± 0.68	93.13 ± 0.69	92.57 ± 0.09	99.4 ± 0.1	>99
Eu	2.41 ± 0.32	1.82 ± 0.07	1.39 ± 0.06	76.6 ± 4.3	58.0
Gd	5.45 ± 0.46	4.26 ± 0.06	4.01 ± 0.09	94.2 ± 2.3	73.6
Tb	0.69 ± 0.01	0.46 ± 0.01	0.43 ± 0.01	93.5 ± 2.7	62.3
Dy	3.54 ± 0.11	2.05 ± 0.03	1.92 ± 0.04	93.6 ± 2.6	54.2
Ho	0.67 ± 0.03	0.35 ± 0.01	0.33 ± 0.01	93.1 ± 2.8	48.6
Er	1.90 ± 0.06	0.98 ± 0.01	0.97 ± 0.03	93.2 ± 2.8	51.2
Tm	0.27 ± 0.03	0.11 ± 0.01	0.10 ± 0.01	92.9 ± 3.0	38.0
Yb	1.65 ± 0.19	0.68 ± 0.01	0.63 ± 0.01	92.9 ± 2.9	38.3
Lu	0.28 ± 0.06	0.09 ± 0.01	0.08 ± 0.01	92.6 ± 3.0	30.0
Pr	10.00 ± 0.21	7.93 ± 0.05	7.76 ± 0.05	97.9 ± 0.7	78.0
Nd	34.15 ± 1.25	22.38 ± 0.09	21.91 ± 0.13	97.9 ± 0.6	64.0
Sm	7.51 ± 0.50	5.98 ± 0.12	2.65 ± 0.07	84.3 ± 2.6	35.3

a Values obtained for DC sample A1.

Except for Eu and Sm, the majority of REEs showed a precipitation above 90%, indicating that under the evaluated conditions, the elements were efficiently separated from the extractor solution. Compared with the total concentration of REEs in DC, a recovery of >90% was achieved for La and Ce. For the other REEs, found at lower concentrations in DC, the recoveries ranged from 30 to 78%. Consequently, after the simple and efficient UAE of these elements from DC, they can be easily recovered, being useful for the industry and contributing to sustainable development.

The results obtained by the proposed process using UAE for extraction and recovery of REEs in DC are in accordance with those reported in the literature. Zhou et al. (2022) studied the kinetics and extraction mechanism of rare earths from Bayan Obo slag using HCl as extractor solvent.^39^ The researchers obtained a high leaching efficiency with HCl 3 mol L^–1^ (>99%). Subsequently, the researchers achieved a greater than 99% REEs recovery with selective precipitation of the elements using oxalic acid.

## 4 Conclusion

The ultrasound-assisted extraction of REEs from DC proved to be simple, low-cost, and highly efficient. The multivariate approach allowed us to obtain the optimized conditions for REEs extraction from DC, providing a high extraction efficiency, especially for La and Ce. The extracted REEs were precipitated as oxalates and, with the exception of Eu and Sm, showed a precipitation efficiency higher than 90%. Ultrasonic extraction and oxalic acid precipitation enabled a recovery of more than 90% for La and Ce, which have the highest concentrations among REEs in the samples, showing the feasibility of recovery of these elements, contributing to sustainable development and circular economy.

## Supplementary Material



## Notes

Major elemental composition of drill cuttings samples determined by energy dispersive X-ray fluorescence (EDXRF), shown in Table S1; concentration of extracted and total REEs in drill cuttings, obtained by ultrasound-assisted extraction and microwave-assisted digestion, presented in Table S2 (PDF)
