# Supplementary material for: Sustainable Ultrasound-Assisted Extraction and Recovery of Rare Earth Elements from Oil and Gas Drill Cuttings
Source: ACS Omega. 2026 Jan 21;11(4):6412–20. doi: 10.1021/acsomega.5c11240 (PMC12878781; doi:10.1021/acsomega.5c11240)
Supplement: Supplementary file 1 [file ao5c11240_si_001.pdf]

Supporting information

Sustainable Ultrasound-Assisted Extraction and Recovery of Rare Earth Elements from Oil and Gas Drill Cuttings

Klaiani Bez Fontana[1], Eduardo S. Chaves[1], Caroline Gonçalves[1], Elias Paiva Ferreira Neto[1,2], Sidney José Lima Ribeiro[2], Rennan Geovanny O. Araujo[3], Tatiane de Andrade Maranhão[1]

[1] *Departamento de Química, Campus Trindade, Universidade Federal de Santa Catarina, 88040-900 Florianópolis, Santa Catarina Brazil*

[2]*Departamento de Química Analítica, Físico-química e Inorgânica, Instituto de Química, Universidade Estadual Paulista, 14800-060 Araraquara, São Paulo, Brazil*

[3] *Departamento de Química Analítica, Instituto de Química, Campus Universitário de Ondina, Universidade Federal da Bahia, 40170-115 Salvador, Bahia, Brasil*

* Email: eduardo.chaves@ufsc.br

Table S1. Major elemental composition of drill cuttings samples determined by energy dispersive X-ray fluorescence (EDXRF).

Samples, %	A1	A2	A3	A4	A5	A6	A7
Na_2O	2.7	3.6	1.8	3.7	3.7	2.4	2.3
MgO	3.6	3.8	3.9	3.9	4.0	3.1	3.5
Al_2O_3	16.4	15.0	17.4	15.3	16.0	15.5	16.2
SiO_2	51.5	53.9	55.2	53.2	53.2	53.5	56.2
K_2O	3.0	3.4	3.3	3.4	3.4	3.4	3.5
Fe_2O_3	5.1	5.3	5.5	5.6	5.8	5.8	5.3
TiO_2	0.8	0.9	0.9	0.8	0.8	0.9	0.8
MnO	0.1	0.1	0.1	0.1	0.1	0.1	0.1
CaO	10.6	9.0	8.2	8.7	8.1	9.9	7.6
BaO	-	0.1	-	0.1	0.2	0.1	-

Table S2. Concentration of extracted and total REEs in drill cuttings, obtained by ultrasound-assisted extraction and microwave-assisted digestion*.

	Concentration, mg kg^{-1}						
**REE**	A1	A2	A3	A4	A5	A6	A7
**La**	42.70 ± 0.33<br>(47.36 ± 0.05)	33.68 ± 0.91<br>(39.84 ± 4.34)	34.09 ± 0.89<br>(41.25 ± 1.81)	36,03 ± 1,01<br>(37.28 ± 0.44)	35.81 ± 0.61<br>(36.11 ± 0.14)	38.97 ± 2.96<br>(39.61± 0.73)	29.30 ± 1.49<br>(26.80 ± 1.14)
**Ce**	96.13 ± 0.69<br>(91.54 ± 0.68)	76,63 ± 1.93<br>(76.48 ± 9.04)	71.88 ± 3.87<br>(76.85 ± 4.13)	80.95 ± 2.02<br>(72.08 ± 1.01)	83.49 ± 1.39<br>(71.35 ± 1.81)	84.82 ± 2.11<br>(77.95 ± 3.77)	61.61 ± 1.23<br>(53.87 ± 1.64)
**Eu**	1.82 ± 0.07<br>(2.41 ± 0.32)	1.29 ± 0.05<br>(1.61 ± 0.20)	1.82 ± 0.04<br>(1.72 ± 0.25)	1.55 ± 0.08<br>(1.57 ± 0.14)	1.65 ± 0.06<br>(1.56 ± 0.11)	1.64 ± 0.05<br>(1.69 ± 0.09)	1.64 ± 0.08<br>(1.43 ± 0.02)
**Gd**	4.26 ± 0.06<br>(5.45 ±0.46)	4.61 ± 0.21<br>(5,24 ± 0,65)	3.77 ± 0.05<br>(5.04 ± 0.56)	4.86 ± 0.02<br>(4.73 ± 0.37)	4.73 ± 0.40<br>(4.84 ± 0.38)	5.02 ± 0.23<br>(5.29 ± 0.56)	4.26 ± 0.28<br>(3.77 ± 0.10)
**Tb**	0.46 ± 0.01<br>(0.69 ±0.01)	0.54 ± 0.02<br>(0.66 ± 0.06)	0.42 ± 0.01<br>(0.63 ± 0.05)	0.56 ± 0.01<br>(0.61 ± 0.03)	0.54 ± 0.06<br>(0.63 ± 0.03)	0.57 ± 0.03<br>(0.68 ± 0.05)	0.49 ± 0.03<br>(0.50 ± 0.02)
**Dy**	2.05 ± 0.03<br>(3.54 ± 0.11)	2.46 ± 0.12<br>(3.34 ± 0.15)	1.86 ± 0.04<br>(3.15± 0.27)	2.50 ± 0.01<br>(3.06 ± 0.19)	2.42 ± 0.32<br>(3.16 ± 0.16)	2.50 ± 0.10<br>(3.41 ± 0.30)	2.16 ± 0.15<br>(2.49 ± 0.09)
**Ho**	0.35 ± 0.01<br>(0.67 ±0.03)	0.40 ± 0.02<br>(0.63 ± 0.02)	0.30 ± 0.01<br>(0.59 ± 0.04)	0.41 ± 0.01<br>(0.58 ± 0.03)	0.40 ± 0.05<br>(0.59 ± 0.03)	0.40 ± 0.02<br>(0.64 ± 0.04)	0.34 ± 0.02<br>(0.47 ± 0.01)
**Er**	0.98 ± 0.01<br>(1.90 ± 0.06)	1.05 ± 0.05<br>(1.78 ± 0.12)	0.82 ± 0.01<br>(1.63 ± 0.14)	1.06 ± 0.01<br>(1,65 ± 0,07)	1.03 ± 0.09<br>(1.66 ± 0.11)	1.06 ± 0.05<br>(1.78 ± 0.15)	0.90 ± 0.01<br>(1.29 ± 0.01)
**Tm**	0.11 ± 0.01<br>(0.27 ± 0.03)	0.11 ± 0.01<br>(0.24 ± 0.01)	0.09 ± 0.01<br>(0.23 ± 0.01)	0.02 ± 0.01<br>(0.22 ± 0.01)	0.11 ± 0.01<br>(0.22 ± 0.01)	0,11 ± 0,01<br>(0.24 ± 0.02)	0,09 ± 0,01<br>(0.18 ± 0.01)
**Yb**	0.68 ± 0.01<br>(1.65 ± 0.19)	0.64 ± 0.03<br>(1.49 ± 0.03)	0.52 ± 0.01<br>(1.42 ± 0.09)	0.67 ± 0.01<br>(1.41 ± 0.07)	0.66 ± 0.03<br>(1.45 ± 0.07)	0.67 ± 0.03<br>(1.52 ± 0.09)	0.54 ± 0.04<br>(1.09 ± 0.05)
**Lu**	0.09 ± 0.01<br>(0.28 ± 0.06)	0.09 ± 0.01<br>(0.26 ± 0.03)	0.07 ± 0.01<br>(0.24 ± 0.02)	0.09 ± 0.01<br>(0.26 ± 0.02)	0.09 ± 0.01<br>(0.25 ± 0.02)	0.09 ± 0.01<br>(0.25 ± 0.02)	0.07 ± 0.01<br>(0.20 ± 0.01)
**Pr**	7.93 ± 0.05<br>(10.0 ± 0.21)	7.56 ± 0.20<br>(8.69 ± 0.77)	6.89 ± 0.17<br>(8.53 ± 0.37)	7.44 ± 0.18<br>(8.07 ± 0.17)	7.56 ± 0.12<br>(8.09 ± 0.08)	7.75 ± 0.24<br>(8.81 ± 0.42)	6.95 ± 0.34<br>(6.20 ± 0.20)
**Nd**	22.38 ± 0.09<br>(34.15 ± 1.25)	22.65 ± 1.11<br>(31.16 ± 3.37)	23.66 ± 1.51<br>(29.70 ± 2.23)	23.85 ± 0.69<br>(28.18 ± 1.57)	23.21 ± 1.24<br>(28.77 ± 1.03)	24.68 ± 0.94<br>(31.66 ± 2.15)	21.06 ± 1.11<br>(21.90 ± 0.53)
**Sm**	5.82 ± 0.12<br>(7.51 ± 0.50)	5.22 ± 0.30<br>(6.11 ± 0.60)	4.88 ± 0.18<br>(6.11 ± 0.54)	5.83 ± 0.19<br>(5.73 ± 0.31)	5.88 ± 0.20<br>(5.72 ± 0.20)	6.04 ± 0.18<br>(6.23 ± 0.46)	5.50 ± 0.30<br>(4.74 ± 0.13)

** Total concentrations obtained after microwave-assisted digestion are parentheses.*
